# FGFR1 expression defines clinically distinct subtypes in pancreatic cancer

**DOI:** 10.1186/s12967-018-1743-9

**Published:** 2018-12-28

**Authors:** Farhan Haq, You-Na Sung, Inkeun Park, Mahmood Akhtar Kayani, Faizah Yousuf, Seung-Mo Hong, Sung-Min Ahn

**Affiliations:** 1 Department of Biosciences, COMSATS University, Islamabad, Pakistan; 2 Department of Pathology, Asan Medical Center, University of Ulsan College of Medicine, Olympic-Ro 43Gil 88, Songpa-Gu, Seoul, Republic of Korea; 3 Division of Oncology, Department of Internal Medicine, Gachon University Gil Hospital, Gachon University Gil Medical Center, Incheon, Republic of Korea; 4 Department of Genome Medicine and Science, College of Medicine, Gachon Institute of Genome Medicine and Science, Gachon University, Seongnam, Republic of Korea

**Keywords:** FGFR1, Classification, Prognosis, Pancreatic cancer, Anti-FGFR1 therapy

## Abstract

**Background:**

The clinical significance of fibroblast growth factor receptor 1 (FGFR1) protein expression in pancreatic cancer is largely unknown. In this study, we aimed investigate the clinical significance of FGFR1 expression in pancreatic cancer.

**Methods:**

First, we investigated the relationship between FGFR pathway gene expression and clinicopathological data in three pancreatic cancer cohorts containing 313 cases. Subsequently, to confirm the findings from the discovery cohorts, we performed immunohistochemistry (IHC) of FGFR1 protein in a validation cohort of 205 pancreatic cancer cases.

**Results:**

In discovery cohort 1, *FGFR1* and Klotho beta (*KLB*) overexpression was associated with low tumor stage (*P* < 0.05), low tumor grade (*P* < 0.05), and better overall survival. Multivariate analysis predicted *FGFR1* (*P *< 0.05) as a prognostic factor for better overall survival. In discovery cohorts 2 and 3, only *FGFR1* overexpression was associated with better overall survival (*P* < 0.05). In the validation cohort, there were 15.7% and 61% strong and weak/moderate FGFR1-positive cases, respectively. FGFR1-positive cases showed better overall survival than FGFR1-negative cases (*P* < 0.05). Furthermore, multivariate analysis revealed FGFR1 positivity as an independent prognostic factor for better overall survival in pancreatic cancer patients (hazard ratio 0.677, 95% confidence interval 0.471–0.972, *P* = 0.035).

**Conclusions:**

FGFR1 expression, as estimated by IHC, may be used to define clinically distinct subtypes in pancreatic cancer. Moreover, FGFR1-based subclassification of pancreatic cancer may lead to new therapeutic approaches for the FGFR1-positive subtype.

**Electronic supplementary material:**

The online version of this article (10.1186/s12967-018-1743-9) contains supplementary material, which is available to authorized users.

## Background

The fibroblast growth factor receptor (FGFR) pathway is one of the major carcinogenic pathways in cancer [1–5]. Genetic deregulation of fibroblast growth factors and their receptors plays an important role in the initiation and progression of different types of cancer [6–9]. Helsten and colleagues [10] reported that the FGFR pathway is the third most frequently altered pathway in cancer, after the p53 and KRAS pathways. Accordingly, cancer drugs targeting the FGFR pathway have been tested in multiple cancers [1, 11]. Currently, phase I and phase II clinical trials of dovitinib, lucitanib, ponatinib, nintedanib, and pazopanib are underway in different solid tumors to block FGFR pathway activation [12–16].

In pancreatic cancer, aberrations in the FGFR pathway, particularly FGFR1 overexpression, have been reported. According to The Cancer Genome Atlas (TCGA) study on pancreatic cancer [17], *FGFR1* is upregulated in approximately 5% of pancreatic cancers. Lehnen and colleagues [18] reported that *FGFR1* was expressed in 4% (5/125) of pancreatic cancer cases, and *FGFR1* amplification was observed in 2.6% (4/155). In contrast, Kornmann and colleagues [19] reported that 57% (4/7) of pancreatic cancer cases showed immunoreactivity for the IIIc splice variant of FGFR1 (FGFR1 IIIc). Nevertheless, the clinical significance of FGFR1 protein expression in pancreatic cancer is still largely unknown.

In this study, we aimed to investigate the clinical significance of FGFR1 overexpression in pancreatic cancer. First, we investigated the clinical significance of FGFR pathway genes using the gene expression and clinicopathological data from three pancreatic cancer cohorts containing 313 cases. Then, to confirm the findings from the discovery cohorts, we performed immunohistochemistry (IHC) targeting FGFR1 protein in a validation cohort of 205 pancreatic cancer cases.

## Methodology

### Data collection and screening

The overall study design is described in Additional file 1: Figure S1. Five FGFR pathway genes that are frequently dysregulated in multiple cancers, namely *FGFR1*, *FGFR4*, *KLB* (an FGFR co-receptor), *FGF19* (the FGFR4 ligand), and *FGF21* (the FGFR1 ligand), were selected for the analysis. Data from discovery cohort 1, consisting of 65 pancreatic cancer patients, were downloaded from the Gene Expression Omnibus database (Accession # GSE62452). Clinical features of discovery cohort 1, including stage, grade, and overall survival information, can be found in Additional file 1: Table S1. LogR expression values of data from the discovery cohort were generated from the Affymetrix Human Gene 1.0 ST array. In discovery cohort 1, the expression status of *FGF19* (probe ID: 7950023), *FGF21* (probe ID: 8030105), *FGFR1* (probe ID: 8150318), *FGFR4* (probe ID: 8110265), and *KLB* (probe ID: 8094679) were screened for the analysis. In discovery cohort 2, LogR expression values were generated using the RSTA Custom Affymetrix 2.0 array (Additional file 1: Figure S1). The expression status of *FGF19* (probe ID: merck-NM_005117_at), *FGF21* (probe ID: merck-NM_019113_at), *FGFR1* (probe IDs: merck-NM_000604_at, merck-NM_023110_a_at, and merck2-NM_001174063.1), *FGFR4* (probe ID: merck-NM_002011_at), and *KLB* (probe IDs: merck-BC033021_at and merck-NM_175737_a_at) was analyzed. In discovery cohort 3, RNA-seq data of 179 pancreatic cancers were analyzed (Additional file 1: Figure S1). The expression of *FGF19*, *FGF21, FGFR1, FGFR4,* and *KLB* was estimated using RNA-seq data with a z-score > 2.0. All detailed information from the pancreatic cancer dataset is available in the public cBioPortal database (Pancreatic Adenocarcinoma, TCGA, provisional).

### Statistical analysis

The associations of *FGF19*, *FGF21, FGFR1, FGFR4,* and *KLB* expression with clinical features, including stage, grade, and survival, were calculated using χ^2^ and Fisher exact tests for the three discovery cohorts. Survival analysis was performed using Kaplan–Meier curves with log-rank (Mantel–Cox) P values. Cox proportional hazard regression and univariate and multivariable analyses were used to evaluate the association between gene expression and survival. Since the sample sizes of the discovery cohorts were small, the multivariable Cox regression model may have led to the overfitting of the data. Therefore, each gene was analyzed separately in combination with the clinical features in multivariable analysis. The hazard ratio (HR) and 95% confidence interval (CI) were also calculated for each factor. P values were two-sided, and *P* < 0.05 was considered to be statistically significant. All statistical analyses were performed with SPSS 21.0 software (IBM, Armonk, NY, USA).

### Validation in 205 patients using IHC

Immunohistochemical labeling was performed in a validation cohort of 205 pancreatic cancer patients at the immunohistochemical laboratory of the Department of Pathology, Asan Medical Center. In brief, 4-μm-thick sections were deparaffinized with xylenes and hydrated in an ethanol series. Endogenous peroxidase activity was blocked by incubation in 3% H_2_O_2_ for 10 min, and then heat-induced antigen retrieval was performed. Primary antibodies were used with a Benchmark autostainer (Ventana Medical Systems, Tucson, AZ, USA) in accordance with the manufacturer’s protocol. Sections were incubated at room temperature for 32 min in primary antibody for FGFR1 (rabbit polyclonal, 1:100; Abnova, Taipei, Taiwan). The sections were then labeled with an automated immunostaining system and processed with an iView DAB detection kit (Benchmark XT, Ventana Medical Systems). Immunostained sections were lightly counterstained with hematoxylin, dehydrated in ethanol, and cleared in xylenes. Immunoreactivity was interpreted by light microscopic examination and independently evaluated by two pathologists, coauthors of this study (Y.N.S. and S.M.H.), who were blind to the clinicopathologic information. Cases were categorized as positive, weak positive and negative.

## Results

### Expression analysis of FGFR genes in discovery cohort 1

The logR expression values of five FGFR-related genes were dichotomized according to their median expressions (Additional file 1: Figure S2). *FGFR1* had the highest expression, with a median enrichment of 5.922, whereas *FGF21* had the lowest expression, with a median enrichment of 2.884. The expression level of *FGFR4* was significantly correlated with those of *FGF19* (Pearson Correlation = 0.30, *P* = 0.014), *KLB* (Pearson Correlation = 0.41, *P* = 0.001), and *FGFR1* (Pearson Correlation = 0.29, *P* = 0.02). Notably, a strong correlation was observed between *FGFR1* and *KLB* expression (Pearson’s correlation = 0.60, *P* < 0.001). *FGF21* expression was not correlated with that of any of the other four genes.

### Association between FGFR genes and clinical features in discovery cohort 1

According to Fisher’s exact test results, overexpression of *FGFR4* (*P* < 0.001) and *KLB* (*P* = 0.005) were significantly associated with a low tumor grade. Overexpression of *FGFR1* was significantly associated with both low tumor grade (*P* = 0.023) and low tumor stage (*P *= 0.023). *FGF19* and *FGF21* overexpression did not show any association with clinical features.

**Fig. 1:**
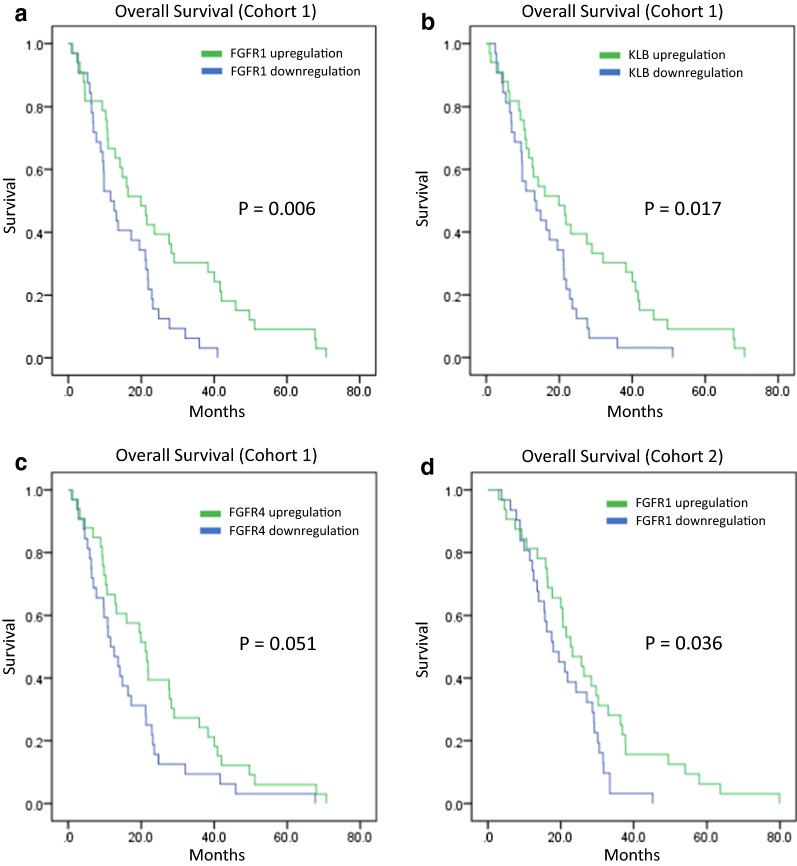
Kaplan–Meier survival analyses of *FGFR1*, *FGFR4,* and *KLB* in pancreatic cancer cohorts 1 and 2. Green represents expression ≥ the median, while blue represents expression < the median. Overall survival by expression of *FGFR1* (**a**), *KLB* (**b**), and *FGFR4* (**c**) in cohort 1. Overall survival by expression of *FGFR1* (**d**) in cohort 2. *FGFR* fibroblast growth factor receptor, *KLB* klotho beta

Next, the association between *FGF19*, *FGF21, FGFR1, FGFR4,* and *KLB* overexpression and the overall survival of pancreatic cancer patients was evaluated. According to univariate analysis, *FGFR1* overexpression (HR 0.475, 95% CI 0.277–0.817, *P* = 0.007) and *KLB* overexpression (HR 0.536, 95% CI 0.318–0.903, *P* = 0.019) were significantly associated with better overall survival. In addition, *FGFR4* overexpression showed a trend toward better overall survival, but it did not reach statistical significance (HR 0.610, 95% CI 0.370–1.008, *P* = 0.054) (Table 1). Kaplan–Meier curves also showed a clear separation between patients with high and low expression of *FGFR1* (Fig. 1a), *KLB* (Fig. 1b), and *FGFR4* (Fig. 1c). Furthermore, multivariable analysis was performed along with the prognostic factors (tumor stage and tumor grade) associated with pancreatic cancer. *FGFR1* expression was the only prognostic factor for better overall survival (HR = 0.524, 95% CI 0.281–0.977, *P* = 0.042) (Table 2).

**Table 1:** Univariate analysis of FGFR-related genes for overall survival in cohort 1

Gene name	Hazard ratio (95% CI)	*P* value
*FGFR1*	0.475 (0.277–0.817)	0.007
*FGFR4*	0.610 (0.370–1.008)	0.054
*FGF19*	1.029 (0.630–1.682)	0.908
*FGF21*	0.853 (0.515–1.413)	0.537
*KLB*	0.536 (0.318–0.903)	0.019

**Table 2:** Multivariate analysis of FGFR-related genes for overall survival in cohort 1

Factors	Hazard ratio (95% CI)	*P* value
*FGFR1*		
FGFR1 (high vs. low)	0.524 (0.281–0.977)	0.042
Tumor grade (1, 2 vs. 3, 4)	0.585 (0.337–1.016)	0.057
Tumor stage (1, 2 vs. 3, 4)	0.814 (0.427–1.548)	0.530
*FGFR4*		
FGFR4 (high vs. low)	0.975 (0.516–1.842)	0.937
Tumor grade (1, 2 vs. 3, 4)	0.514 (0.263–1.002)	0.051
Tumor stage (1, 2 vs. 3, 4)	1.109 (0.614–2.001)	0.732
*KLB*		
KLB (high vs. low)	0.640 (0.375–1.093)	0.102
Tumor grade (1, 2 vs. 3, 4)	0.547 (0.316–0.945)	0.031
Tumor stage (1, 2 vs. 3, 4)	1.082 (0.606–1.931)	0.791

### Expression and survival analysis of FGFR genes in discovery cohort 2

The potential significance of the FGFR-pathway genes was further evaluated in cohort 2. Similarly, logR expression values of the five FGFR-related genes were dichotomized according to their medians (Additional file 1: Figure S3). Consistent with the results in cohort 1, the highest median expression was observed for *FGFR1*. Of note, of all five genes, only patients with *FGFR1* expression had significantly better overall survival by Kaplan–Meier analysis (Fig. 1d).

### Expression and survival analysis of FGFR genes in discovery cohort 3

The TCGA pancreatic cancer dataset, which includes data from 179 pancreatic cancer patients, was selected as cohort 3. Overexpression of *FGFR1, KLB, FGF19, FGFR4,* and *FGF21* was identified in 7 (4%), 7 (4%), 6 (3%), 5 (2.8%), and 2 (1.1%) patients, respectively (Additional file 1: Figure S4A). Notably, only *FGFR1* overexpression was significantly associated with better overall (*P* = 0.0158) and disease-free survival (*P* = 0.006) (Additional file 1: Figure S4B).

### FGFR1 immunolabeling in the validation cohort

**Fig. 2:**
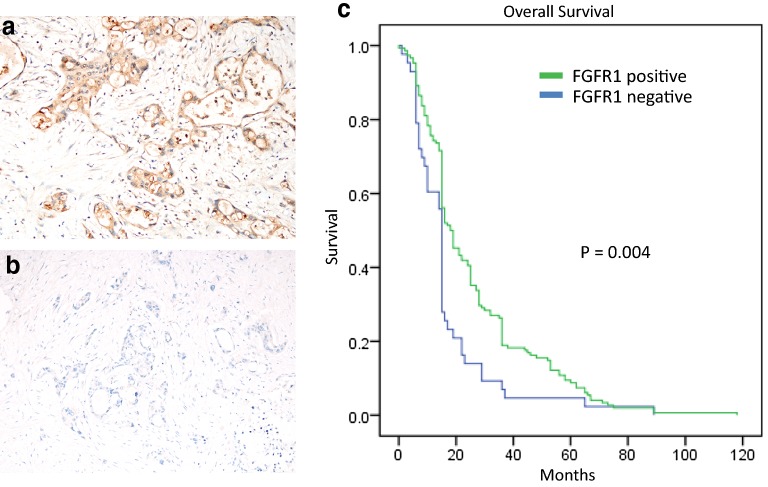
Immunohistochemistry and Kaplan–Meier survival analysis of FGFR1 in the validation cohort of 205 pancreatic cancer patients. **a**, **b** Represent immunohistochemistry results of FGFR1-positive and -negative cases, respectively. **c** Overall survival of pancreatic cancer patients in the discovery cohort with high and low FGFR1 (**c**). FGFR: fibroblast growth factor receptor

Representative IHC images of FGFR1-positive and -negative cases from the validation cohort are depicted in Fig. 2a, b, respectively. Thirty (15.7%) cases were strongly FGFR1-positive, and 118 (61%) were moderately/weakly positive. Forty-three cases (22.5%) were negative for FGFR1 expression.

### Univariate and multivariate analysis for overall survival

According to the statistical analysis, no significant associations were observed between FGFR1 positivity and poor clinicopathological features, including bile duct invasion, duodenal invasion, perineural invasion, lymphovascular invasion, and lymph node metastasis. However, in univariate analysis, overall survival was significantly associated with age (HR 1.451, 95% CI 1.073–1.961, *P* = 0.016), bile duct invasion (HR 1.469, 95% CI 1.099–1.963, *P* = 0.009), lymphovascular invasion (HR 1.455, 95% CI 1.088–1.944, *P* = 0.011), lymph node metastasis (HR 2.495, 95% CI 1.811–3.436, *P* < 0.001), and FGFR1 expression (HR 0.590, 95% CI 0.415–0.839, *P* = 0.003) (Table 3). Furthermore, in the multivariate analysis, age (HR = 1.587, 95% CI 1.130–2.230, *P* = 0.008), bile duct invasion (HR 1.657, 95% CI 1.211–2.269, *P* = 0.002), lymph node metastasis (HR 2.360, 95% CI 1.665–3.344, *P* < 0.001), and *FGFR1* expression (HR 0.677, 95% CI 0.471–0.972, *P* = 0.035) were predicted as significant prognostic biomarkers for overall survival in pancreatic cancer. Notably, FGFR1 expression was the only prognostic biomarker for better overall survival (i.e., HR < 1) (Table 3).

**Table 3:** Cox regression analysis for overall survival in 205 pancreatic cancer patients

	*P* value	HR	Univariate Analysis		*P* value	HR	Multivariate analysis	
			95.0% CI for HR				95.0% CI for HR	
			Lower	Upper			Lower	upper
Age, years (≥ 60 vs. < 60)	0.016	1.451	1.073	1.961	0.008	1.587	1.130	2.230
Gender	0.235	1.191	0.892	1.591				
Tumor size, cm (≥ 2 vs. < 2)	0.241	1.534	0.751	3.135				
Differentiation (well/moderate vs. poor)	0.076	1.428	0.964	2.115				
Extension (confined vs. peripancreatic soft tissue)	0.061	1.898	0.970	3.714				
Bile duct invasion (absent vs. present)	0.009	1.469	1.099	1.963	0.002	1.657	1.211	2.269
Duodenum invasion (absent vs. present)	0.074	1.303	0.975	1.742				
Lymphovascular invasion (absent vs. present)	0.011	1.455	1.088	1.944	0.098	1.320	.950	1.834
Perineural invasion (absent vs. present)	0.086	1.403	.953	2.064				
Lymph node metastasis (absent vs. present)	< 0.001	2.495	1.811	3.436	< 0.0001	2.360	1.665	3.344
Tumor stage^a^	0.059	1.382	0.988	1.933				
FGFR1 (positive vs. negative)	0.003	0.590	0.415	0.839	0.035	0.677	0.471	0.972

^a^Tumor stage was not included for multivariate analysis because tumor size, in addition to bile duct and duodenal invasion and extension (confined vs peripancreatic soft tissue extension), are components of T stage

## Discussion

The main finding of this study is that FGFR1 protein expression defines clinically distinct subtypes of pancreatic cancer. FGFR1-positive cases showed better overall survival than FGFR1-negative cases. To the best of our knowledge, this study is the first to demonstrate the clinical significance of FGFR1 overexpression in pancreatic cancer.

The clinical significance of *FGFR1* overexpression has not been thoroughly investigated in pancreatic cancer for two reasons: (1) *KRAS* mutation, which does not have any targeted solution, is almost universal in pancreatic cancer; (2) *FGFR1* overexpression is not exclusive with *KRAS* mutation [17, 20]. Although, recent genomic and transcriptomic studies identified new subtypes of PADC, but prognostic role of *FGFR1* is not highlighted. For instance, Bailey and colleagues demonstrated that *Kras*^*G12D/*+^*;Trp53*^*fl/*+^ mutant subtype (containing group of genes with *FGFR1* expression) showed less aggressive clinical outcome compared to *Kras*^*G12D/*+^*;Trp53*^*fl/*+^*;TAp63*^*fl/fl*^ mutant subtype (containing group of genes without *FGFR1* expression) in pancreatic cancer [21]. Furthermore, another transcriptomic profiling study classified PADC patients into L1–L6 subtypes. Interestingly, the L5 subtype which showed most favorable clinical outcome from the rest of the molecular subtypes was also enriched with FGFR1 pathway related genes [22].

Our finding can have immediate clinical ramifications. In pancreatic cancer, FGFR1 is the most frequently altered receptor of the four FGFR receptors, and, using readily applicable immunohistochemistry methods, FGFR1 can be used to classify pancreatic cancer into FGFR1-positive and -negative subtypes. As FGFR1-positive pancreatic cancer has better prognosis, FGFR1 can be used as an independent predictor of better overall survival in pancreatic cancer patients. Moreover, FGFR1-based subclassification of pancreatic cancer may lead to new therapeutic approaches for the FGFR1-positive subtype. For example, it may be possible to target FGFR1 using antibody in strongly FGFR1-positive pancreatic cancer, in the same manner in which HER2 is targeted using anti-HER2 antibodies in HER2-positive breast cancer.

Only a few previous studies have examined *FGFR1* expression in pancreas cancers, and they reported a wide range of FGFR1 positivity (4–57%) [18, 19, 23]. Lehnen and colleagues observed that 4% (5/125) of pancreatic cancer patients showed FGFR1 expression, and amplification was noted in 2.6% of the cases (4/155) [18]. In contrast, Kornmann et al. reported that 57% (4/7) of pancreas cancers showed immunoreactivity for FGFR1 IIIc [19]. Our study is unique in that we evaluated FGFR1 expression based on the intensity of FGFR1 labeling, and we observed strong FGFR1 positivity in 15.7% (30 cases) and moderate/weak FGFR1 positivity in 61.8% (118 cases) in a cohort of Korean pancreatic cancer patients. Using only the strong FGFR1 expression group, the frequency of FGFR1 labeling was higher than the results of Lehnen and colleagues’ study. However, if we include moderate/weak FGFR1 labeling, the frequency was similar to that of Kornmann’s study. This wide range of FGFR1 labeling results may be plausibly explained by different FGFR1 expression rates based on different ethnic groups (Korean and Western populations), use of different antibody clones, or different cutoffs for FGFR1 labeling.

## Conclusion

In summary, FGFR1 overexpression, evaluated by IHC, may be used as a prognostic biomarker for overall survival in pancreatic cancer patients. Moreover, FGFR1 overexpression may define a subset of pancreatic cancer, leading to new therapeutic approaches.

## Additional file

**Additional file 1.** Additional figures and table.
