# Supplementary material for: FGFR1 expression defines clinically distinct subtypes in pancreatic cancer
Source: J Transl Med. 2018 Dec 28;16:374. doi: 10.1186/s12967-018-1743-9 (PMC6311038; doi:10.1186/s12967-018-1743-9)
Supplement: Supplementary file 1 — Additional file 1. Additional figures and table. [file 12967_2018_1743_MOESM1_ESM.docx]

**FGFR1 expression defines clinically distinct subtypes in pancreatic cancer**

Farhan Haq^*1^, You-Na Sung*^2^, Inkeun Park^3^, Mahmood Akhtar Kayani^1^, Faiza Yousuf^1^, Seung-Mo Hong^2#^, Sung-Min Ahn^3,4#^

^1^Department of Biosciences, COMSATS University, Islamabad, Pakistan

^2^Department of Pathology, Asan Medical Center, University of Ulsan College of Medicine, Seoul, Korea

^3^Division of Oncology, Department of Internal Medicine, Gachon University Gil Hospital, Incheon, South Korea

^4^Department of Genome Medicine and Science, College of Medicine, Gachon University, Seongnam, South Korea

^*^These authors contributed equally to the study

^#^Correspondence to:

**Sung-Min Ahn, M.D., PhD**

Div. of Oncology, Dept. of Internal Medicine

Dept. of Genome Medicine and Science

Gachon Institute of Genome Medicine and Science

Gachon University Gil Medical Center

smahn@gachon.ac.kr/ ahnsungmin@gmail.com

032-460-2177 / 010-3648-7437

**Seung-Mo Hong M.D., PhD**

Department of Pathology,

Asan Medical Center,

University of Ulsan College of Medicine,

Olympic-Ro 43Gil 88, Songpa-Gu, Seoul,

smhong28@gmail.com

82-2-3010-4558

**Additional Material**

**Figure S1.** Over workflow of the study.

**Figure S2:** LogR expression values of *FGFR* pathway genes in cohort 1

**Figure S3:** LogR expression values of *FGFR* pathway genes in cohort 2

**Figure S4:** Expression and Kaplan Meier Analysis of *FGFR* pathway genes in cohort 2. A) The frequency of overexpression of *FGFR1*, *KLB*, *FGF19*, *FGFR4* and *FGF21* according z-score is shown. Only *FGFR1* overexpression was associated with better overall (B) and disease-free survival (C) of pancreatic cancer patients.

**Table S1:** Clinicopathological features of 65 pancreatic cancer patients of cohort 1


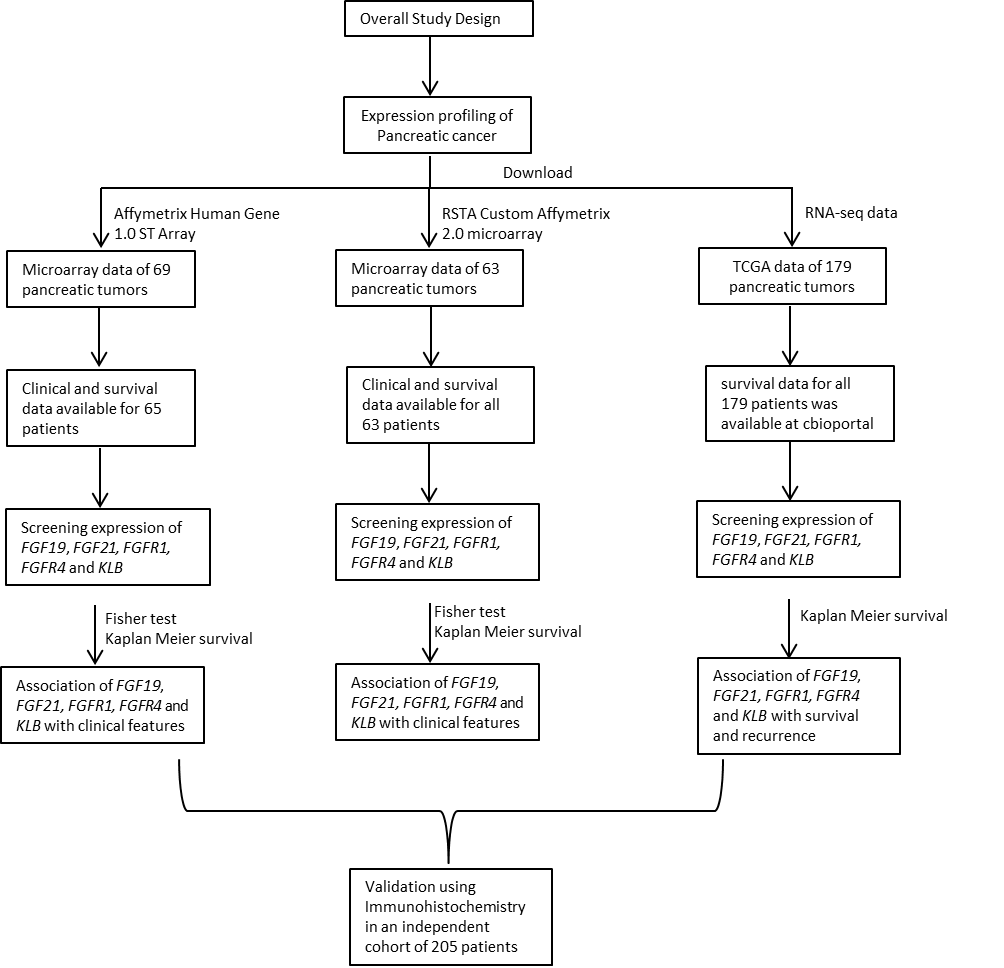


**Figure S1**

**
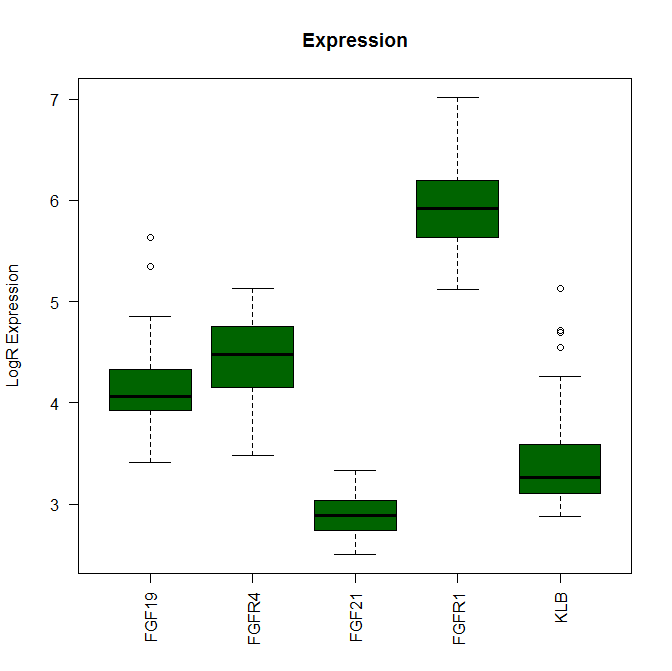
**

**Figure S2**

**
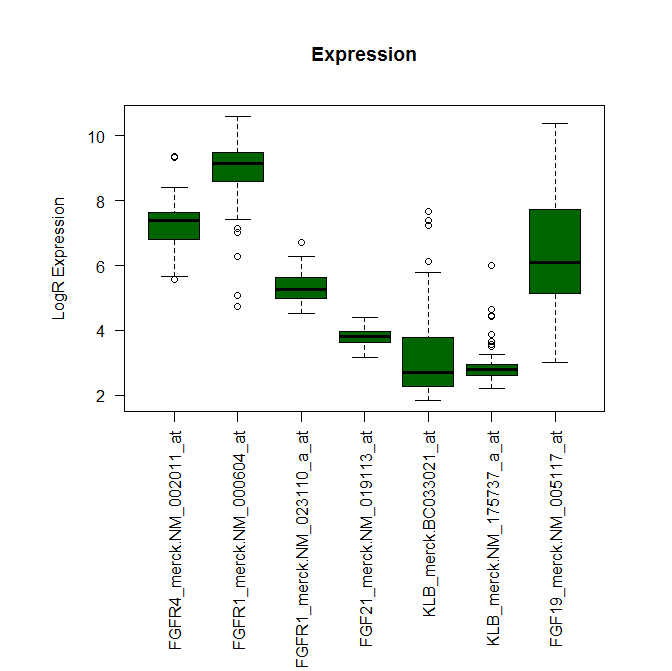
**

**Figure S3**

**
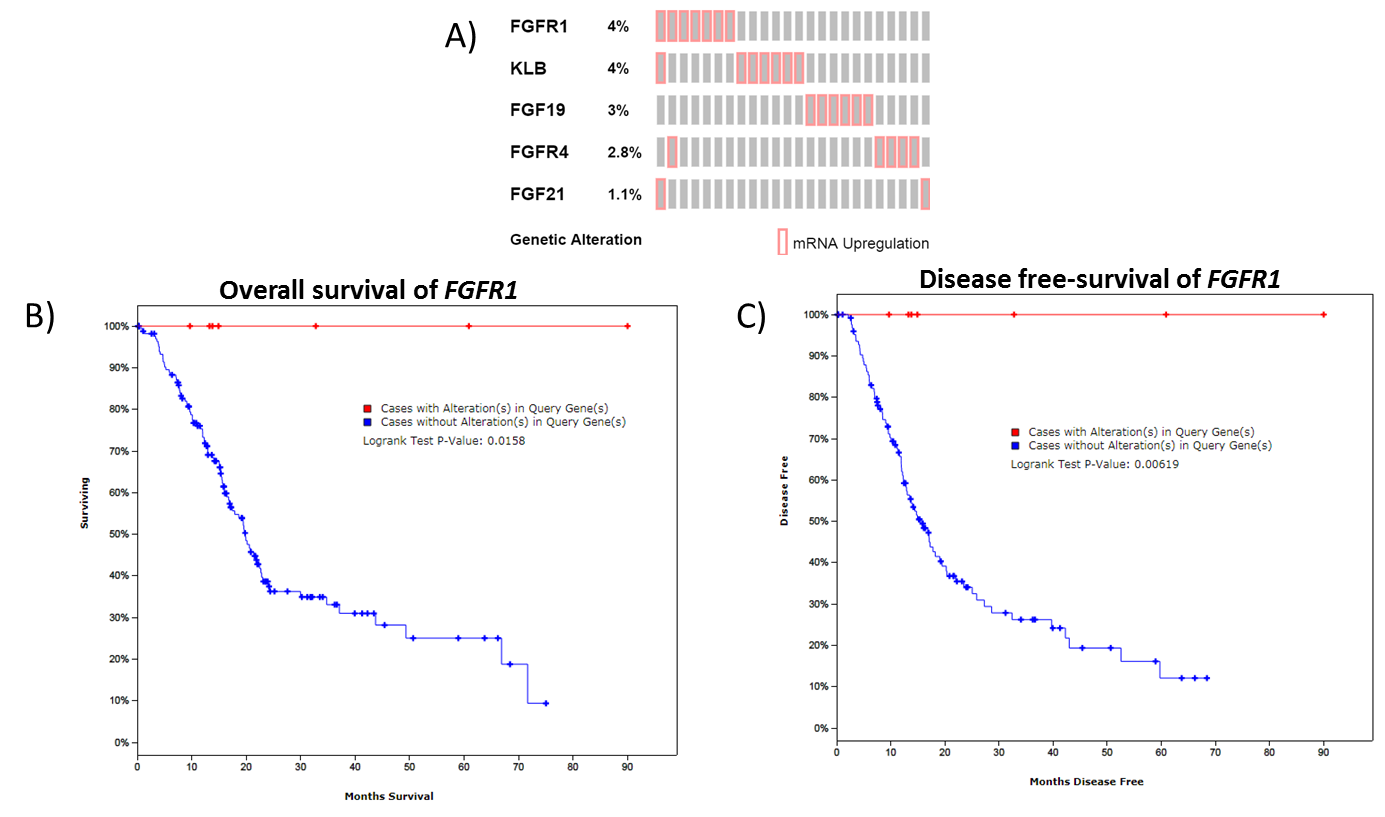
**

**Figure S4**

**Table S1**

| **Total patients** | 65 |
| --- | --- |
| **Tumor stage** |  |
| **Stage 1** | 4 (6.15%) |
| **Stage 2** | 45 (69.23%) |
| **Stage 3** | 10 (15.38%) |
| **Stage 4** | 6 (9.23%) |
| **Tumor grade** |  |
| **Grade 1** | 2 (3.07%) |
| **Grade 2** | 32 (49.23%) |
| **Grade 3** | 29 (44.61%) |
| **Grade 4** | 1 (1.53%) |
| **NA** | 1 (1.53%) |
| **Mortality status** |  |
| **Dead** | 49 (75.38%) |
| **Alive** | 16 (24.62) |
